# Completing a molecular timetree of apes and monkeys

**DOI:** 10.3389/fbinf.2023.1284744

**Published:** 2023-12-15

**Authors:** Jack M. Craig, Grace L. Bamba, Jose Barba-Montoya, S. Blair Hedges, Sudhir Kumar

**Affiliations:** ^1^ Department of Biology, Temple University, Philadelphia, PA, United States; ^2^ Institute for Genomics and Evolutionary Medicine, Temple University, Philadelphia, PA, United States; ^3^ Center for Biodiversity, Temple University, Philadelphia, PA, United States

**Keywords:** monkeys, phylogeny, dating, imputation, taxonomy

## Abstract

The primate infraorder Simiiformes, comprising Old and New World monkeys and apes, includes the most well-studied species on earth. Their most comprehensive molecular timetree, assembled from thousands of published studies, is found in the TimeTree database and contains 268 simiiform species. It is, however, missing 38 out of 306 named species in the NCBI taxonomy for which at least one molecular sequence exists in the NCBI GenBank. We developed a three-pronged approach to expanding the timetree of Simiiformes to contain 306 species. First, molecular divergence times were searched and found for 21 missing species in timetrees published across 15 studies. Second, untimed molecular phylogenies were searched and scaled to time using relaxed clocks to add four more species. Third, we reconstructed ten new timetrees from genetic data in GenBank, allowing us to incorporate 13 more species. Finally, we assembled the most comprehensive molecular timetree of Simiiformes containing all 306 species for which any molecular data exists. We compared the species divergence times with those previously imputed using statistical approaches in the absence of molecular data. The latter data-less imputed times were not significantly correlated with those derived from the molecular data. Also, using phylogenies containing imputed times produced different trends of evolutionary distinctiveness and speciation rates over time than those produced using the molecular timetree. These results demonstrate that more complete clade-specific timetrees can be produced by analyzing existing information, which we hope will encourage future efforts to fill in the missing taxa in the global timetree of life.

## 1 Introduction

With the global biodiversity crisis threatening species worldwide, the work of taxonomists and systematists to catalog Earth’s species is critically important (Singh, 2002; Albert et al., 2021). There are more than two million species extant on earth, but only 5% of which represent unique species-level taxa (Kumar et al., 2022) in the largest online taxonomic database (NCBI Taxonomy) with molecular sequences (Schoch et al., 2020; Kumar et al., 2022). Less than a third of these species are included in the largest global dated phylogeny based on genetic data, TimeTree (version 5, hereafter TT5) (Kumar et al., 2022). If we are to have any hope of conserving Earth’s diminishing biodiversity, we must find a way to build a global tree of life to better understand species relationships and their divergence times.

Yet taxonomically complete, large-scale phylogenies of species are still not available for even widely studied groups. Many researchers rely on phylogenetic imputation using polytomy resolvers (Thomas et al., 2013; Chang et al., 2020; Kumar et al., 2022) to fill gaps in their phylogenies, but these approaches are susceptible to biases that may undermine downstream analyses (Weedop et al., 2019). As the inference of historical processes from a phylogeny is already difficult (Louca and Pennell, 2020; Craig et al., 2022; Louca et al., 2022), an approach to large-scale phylogenetics with reduced reliance on data-less phylogenetic imputation would go a long way to resolving this ambiguity.

Primates represent an ideal test case for such an approach, as they have consistently and frequently been the subject of divergence time analysis. The initial studies on Simiiformes emerged at the onset of molecular clock research (Sarich and Wilson, 1967; Uzzell and Pilbeam, 1971), and this interest has persisted in multigene studies of many species ((Sarich and Wilson, 1967; Read and Lestrel, 1970; Uzzell and Pilbeam, 1971; Lovejoy et al., 1972; Kumar and Hedges, 1998), and phylogenomic studies comprising hundreds of primate species (Reis et al., 2018; Kuderna et al., 2023). Here, we relied on the NCBI Taxonomy database (Schoch et al., 2020) as a taxonomic reference and explored leveraging this wealth of readily available genetic and phylogenetic data to expand the phylogeny of apes and monkeys (Simiiformes) to contain all 306 species for which molecular data exist in the GenBank.

Taking the large-scale synthetic TT5 (Kumar et al., 2022), derived from 4,185 published time-calibrated genetic phylogenies as a backbone, we explored different means to incorporate missing species by searching for published timetrees, untimed molecular phylogenies, and genetic data available through GenBank (Clark et al., 2016). We present our approaches to carrying out these three types of analyses and report an expanded timetree (eTT) of all 306 living simiiform species containing at least one molecular sequence in the NCBI taxonomy database tied to GenBank.

This description is followed by comparing the molecular divergence times computed in this study with those obtained by statistical imputations reported in the VertLife resource (Upham et al., 2019). Moreover, we compared the patterns of evolutionary isolation (Redding, et al., 2014), a common tool for identifying conservation priorities, and inferences of speciation rates, obtained by using our timetree and that containing dataless times because these metrics may be influenced by dataless imputations (Weedop et al., 2019; Craig et al., 2022).

## 2 Results

### 2.1 The expanded timetree of Simiiformes

We began with the NCBI taxonomy (Schoch et al., 2020) of 308 simiiform species as our reference for the purposes of this study, with the goal of building an expanded timetree of all species represented by molecular data. Of these, two species, *Cheracebus medemi* Hershkovitz, 1963 and *Callicebus oenanthe* Thomas 1924, had no molecular sequences in GenBank at the time of this writing. Therefore, up to 38 species could be added to TT5 to form an expanded timetree (eTT).

**FIGURE 1:**
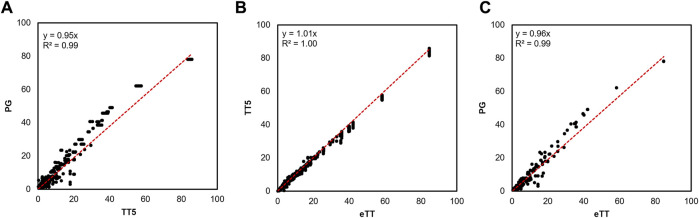
Comparison of the ages of shared nodes between timetrees. **(A)** TT5 and the PG timetree share comparable node times (*R* ^2^ = 0.99) with only slightly higher divergence times in the PG tree (slope = 0.95). **(B)** TT5 is closely correlated with our expanded timetree (eTT) (*R* ^2^ = 1.0), with nearly no bias towards older or younger times between them (linear regression slope = 1.01). **(C)** The PG phylogeny of Kuderna et al. (2023) (Kuderna et al., 2023) is also strongly correlated with our eTT (*R* ^2^ = 0.99), with a slight bias towards older times (linear regression slope = 0.96), as was the case when comparing this tree to TT5.

In the first step, we manually searched the corpus of published articles reporting molecular timetrees may contain these missing species (Section 4). This search yielded 15 published studies, including a substantial phylogenomic timetree (Kuderna et al., 2023) (henceforth PG timetree) containing 8 missing simiiform species. Nodes shared between TT5 and the state-of-the-art PG timetrees were highly concordant in divergence time (Figure 1A, linear regression slope = 0.95, *R* ^*2*^ = 0.99). This close relationship suggests that the TT5 and PG timetrees are in agreement, validating the choice of TT5 as a backbone. We used the HAL approach (Hedges et al., 2015) to combine the TT5 and PG timetrees to produce an expanded simiiform backbone timetree containing 276 of 306 species. As expected, the resulting timetree was highly concordant with its constituent timetrees as the regression slope is close to 1.0 (Figures 1B, C).

**FIGURE 2:**
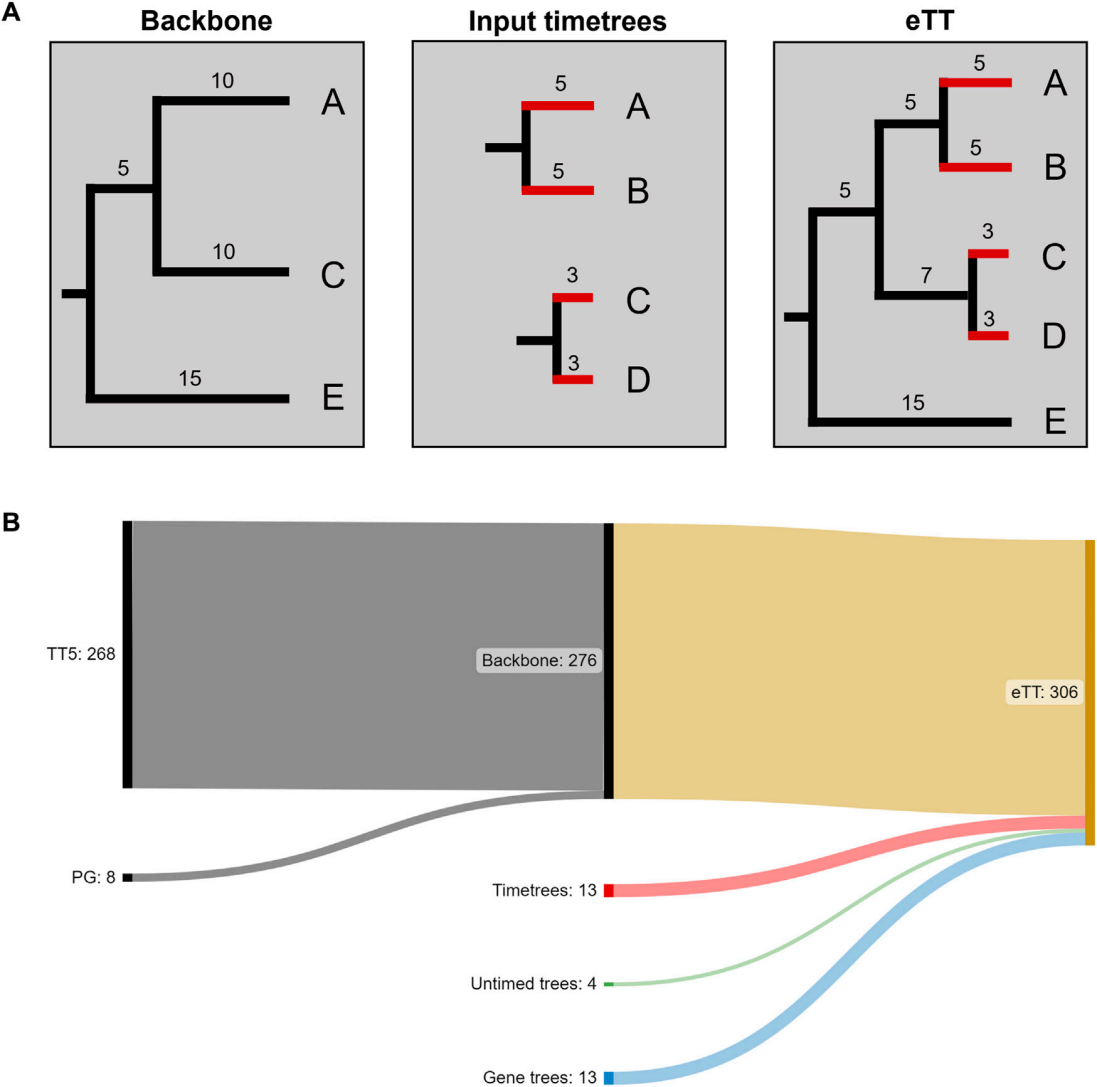
Assembling the expanded timetree (eTT). **(A)** The backbone phylogeny comprises species A, C and E. We then identify pendant lengths (in red) and sister species (A, C) for the missing species B and D based on a set of input timetrees. We finally combine these with the backbone to create the eTT. **(B)** Sankey plot of the numbers of species included from different data sources.

We found 13 further missing species in published timetrees. From these phylogenies, we identified the sister species and terminal branch lengths (pendant lengths, PL, in units of millions of years) and added them to the eTT. Phylogenetic trees containing four other missing species were found in the literature in publications that did not carry out molecular dating analyses. We converted these phylogenies with branch lengths into timetrees using secondary calibration times derived from the TimeTree database (Figure 2A). With these species, the eTT expanded to 293 species (Figure 2B).

**FIGURE 3:**
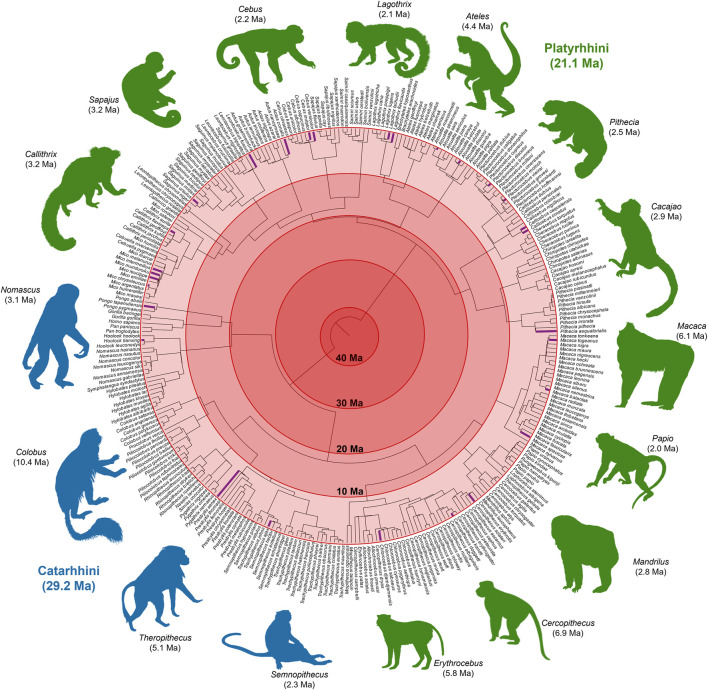
A molecular timetree of 306 species of Simiiformes (apes and monkeys). This phylogeny was constructed by taking simiiform species from TT5, the largest supertree of living organisms available, and expanding it using additional published phylogenies, both timed and untimed, and genetic data on GenBank to infer the topology and branch lengths for the missing species. From a backbone of 268 apes and monkeys, we added 38 additional species (shown in purple) for a total of 306 species. Images from Phylopic.org. Phylogeny in Newick format is available in Supplementary Material.

For 13 more missing species, GenBank contained the sequences of mostly mitochondrial genes. We built timetrees using sequences from these species and their close congeners (Section 4). Integrating these yielded the most comprehensive timetree of Simiiformes to date, comprising all living species of apes and monkeys with any molecular data in GenBank (Figure 3).

### 2.2 Comparing dataless imputation of divergence times with molecular-derived estimates

We compared node times in the expanded timetree of Simiiformes, based on molecular data exclusively, with those derived from dataless phylogenetic imputation available from the VertLife resource. The VertLife phylogeny is a tree of 5,911 mammal species of which 1,813 (30.1%) were added by phylogenetic imputation (Upham et al., 2019). The age of the common ancestor of Simiiformes in the VertLife timetree was 32.7 million years (myr), which is younger than 39.0 myr (36.7–41.4) inferred in the PG timetree (Kuderna et al., 2023) and 43.0 myr (40.0–44.2) in TT5. The discordance in crown times across studies is likely due to differing calibration schemes (Hedges et al., 2018).

**FIGURE 4:**
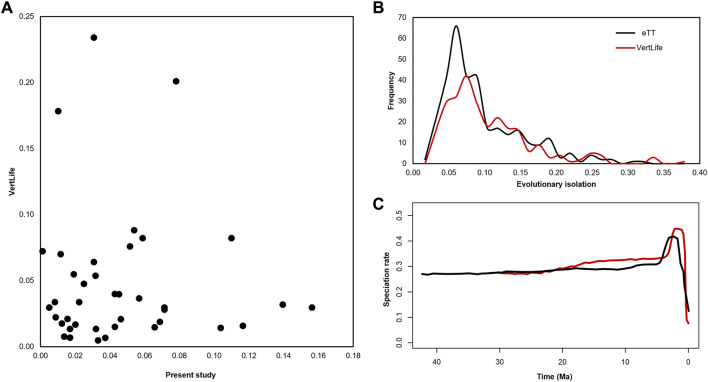
**(A)** Pendant lengths (PL), the lengths of the terminal branches subtending each tip, differ substantially when comparing the 39 tips added by imputation to the VertLife (Upham et al., 2019) phylogeny of apes and monkeys and the same tips in our phylogeny, even when accounting for differences in the crown age of each phylogeny. There is virtually no correlation between the two sets of times (*R* ^2^ = 0.00). **(B)** When we normalize evolutionary isolation (EI) by the crown age of the phylogeny, which makes it inter-compatible between trees, we find that EI, which measures the amount of unique evolutionary history captured by each species, is roughly normally distributed with a long right tail of highly distinct species characterized by substantial unique evolutionary history in our phylogeny (black). By contrast, the values observed from the VertLife phylogeny (red), even when scaling by the total crown age of the phylogeny, are consistently lower, especially for the more common, lower-EI species. **(C)** In our eTT we find the rate of speciation across apes and monkeys through time is relatively constant, with the exception of a terminal artifact typically ascribed to uncataloged taxonomic diversity.

So, we scaled all the divergence times in our 306 species eTT and the VertLife timetree by dividing them by their respective crown ages (42.3 myr and 32.7 myr, respectively). A comparison of only the imputed (scaled) pendant lengths (PLs) in the VertLife timetree (excluding those derived from genetic data) with those in our strictly molecular eTT (Figure 4A), clearly showed a lack of relationship (*R* ^2^ ∼ 0.0). That is, divergence times for the tip taxa derived from dataless imputation and genetic sequence data are quite different. Importantly, 20 of 39 of the taxa (74.6%) that were imputed in the VertLife tree were incorporated into our tree from either TT5 or the PG timetree, not as part of the present study, making it unlikely that our approach influenced this pattern. Therefore, molecular data provide fundamentally different time estimates than those imputed without molecular data, regardless of the phylogenetic approach.

The predominant reason for the lack of correlation is the taxonomic discrepancy between the trees, in which the branching orders of some clades may differ. Of those cases in which the molecular and imputed time estimates differ by more than 1 myr, molecular data place some species at the root of the genus, whereas VertLife has them nested within their genus, which resulted in underestimates of time in the VertLife tree for five species. The reverse is true for two further species, where VertLife overestimated time. In two cases, molecular data suggest paraphyletic genera, whereas VertLife presents them as monophyletic. Notably, the greatest of these discrepancies is only 6.1 myr, less than a quarter of the crown age of the tree, as only tip taxa were compared.

### 2.3 Impact of dataless imputation of divergence times on evolutionary isolation and speciation rates

The differences we observed between the eTT and the imputed VertLife timetree had some effect on the downstream analysis. We found that the equal splits statistic of evolutionary isolation (EI) (Redding et al., 2014), which partitions the total phylogenetic diversity into the amount of unique evolution represented by each species in years, exhibited a left-skewed distribution in our timetree (Figure 4B). While most species are members of highly diverse clades like *Macaca* (average EI = 3.01 myr) and, therefore, are characterized by low levels of unique evolutionary history, there remains a long right tail of species in less diverse clades representing millions of years of more unique evolution, such as *Homo sapiens* (EI = 11.33 myr). This overall pattern is true of the VertLife tree as well, but the normalized VertLife EI values are lower overall, especially for the left side of the distribution, comprising more common lower-EI species. In other words, even accounting for the difference in crown ages, VertLife consistently underestimates the evolutionary isolation of many species, which inevitably impacts any downstream analyses that treat EI as a determining factor in, for example, determining conservation priorities.

In addition, we inferred the pattern of speciation through time across the whole timetree of Simiiformes using a Bayesian framework (Höhna et al., 2016). Except for the terminal artifact known to be associated with taxonomic shortfall, either due to undescribed species or known species missing from the phylogeny (Craig et al., 2022), we recover a near-constant clade-wide rate of speciation (Figure 4C). The same clock-like pattern holds true of the VertLife phylogeny, where there is a higher amplitude terminal artifact extending further into the present (Marin and Hedges, 2018; Craig et al., 2022). Therefore, we find support for the hypothesis that when analyzing taxonomically rich timetrees at a large enough phylogenetic scale (hundreds of species across higher taxonomic ranks), the prevailing macroevolutionary trend is “clock-like” speciation rate constancy (Hedges et al., 2015). It has been proposed that new species arise as a result of biogeographic events such as orogeny and river capture, and as these events are ultimately stochastic at a large enough time scale, the resulting pattern of speciation is effectively random and appears constant throughout time (Hedges et al., 2015).

## 3 Conclusion

Access to species phylogeny is extremely useful in identifying conservation priorities (Gumbs et al., 2017), as global biodiversity is under unprecedented threat (Estrada et al., 2017). Remarkably, much of the data we need to resolve significant questions amid the ongoing biodiversity crisis may already exist in some cases, and are simply awaiting a novel synthetic approach to integrate them into a phylogenetic consensus. Furthermore, these newly emerging data-rich synthetic phylogenies give us the potential to revisit major macroevolutionary questions, such as the overall pattern of speciation through time (Hedges et al., 2015; Craig et al., 2022).

We have shown that the state of knowledge of the sequence data availability enables us to create far more complete phylogenies than we currently have while avoiding the potential biases incurred by phylogenetic imputation (Weedop et al., 2019). We propose that it is possible to synthesize and analyze the vast amount of genetic and phylogenetic data already available into more comprehensive expanded timetrees (eTTs) across the tree of life, leading to a better understanding of evolutionary processes and better protection for species at risk. By using the approach we outline here, researchers will be able to construct new taxonomically complete eTTs without the potential statistical artifacts attributable to dataless phylogenetic imputation.

## 4 Methodological details

### 4.1 Taxonomic reference

We took the NCBI taxonomy database (Schoch et al., 2020) as our taxonomic reference for the purposes of this study. While different taxonomic disciplines maintain their own species lists, and our approach is equally compatible with any of these, the NCBI taxonomy database is frequently updated and directly tied to the GenBank repository for sequence data, making it the optimal reference source as our focus is on timetrees derived from the molecular data. After removing all unidentified and uncategorized samples, redundant subspecies, populations, strains, or other sub-specific taxa (keeping only the type population wherever possible), two species without any sequences, and the extinct species *Homo heidelbergensis*, there were 306 simiiform species-level taxa present in the NCBI taxonomy database. Most of these include a published article, from which we could extract a phylogeny or a link to sequence data on GenBank.

### 4.2 Backbone phylogeny

We assembled a simiiform backbone using two large-scale timetrees. TimeTree (Hedges et al., 2015) is a supertree built from 4,185 published timed phylogenies, currently including 148,876 species. Timetree includes 268 of 306 simiiform species with molecular data in the NCBI taxonomy database. We expanded this backbone by combining it with the largest phylogenomic tree of primate species to date, Kuderna et al. (2023), containing over 80% of primate genera. It includes 155 simiiform species, eight of which were absent in TT5. We combined these two phylogenies using the HAL approach that was used to build TT5 (Hedges et al., 2015; Kumar et al., 2022).

### 4.3 Phylogenies of missing simiiform species

There were 30 simiiform species present in the NCBI taxonomy database but not our backbone phylogeny, falling into three categories. First, 13 species were present in published, timed phylogenies in 12 articles. Second, four species were present in four published phylogenies that had not been subjected to relaxed clock dating to obtain timetrees. Third, 13 species were available only as published sequence data on GenBank, often with only a single protein or gene available, such as the common barcoding gene *CO1* or *Cyt-B*.

For the first category of missing species, for which timetrees are available, we accessed these timetrees through the materials provided for publication (2 timetrees) or by manual reproduction from published figures (11 timetrees). These timetrees were selected based on using modern phylogenetic methods and exclusively molecular data. In all cases where multiple representatives of the same species were included, we retained a holotype or paratype wherever possible, or a specimen sampled near the type locality. We then noted the sister species and terminal branch length (pendant length, PL) for each species missing from our backbone timetree, and used this information to add them into the final timetree. This approach allows the incorporation of published divergence times derived from genetic data into an existing tree without using any special algorithms. In cases where the PL of species derived from published studies exceeded those of their sister species in our backbone, we set both of their PLs to that of the backbone species, with a negligible offset value of 0.1 to avoid polytomies. This was necessary for nine nodes; the difference was usually small (median 0.2 myr). In one case (*Lagothrix flavicauda*), taxonomic uncertainty precluded the designation of a consensus sister species, so we placed the new species at the base of the genus.

For species included in published phylogenies that were not time-calibrated, we accessed the appropriate phylogenies as before and then time-calibrated them using literature consensus calibrations derived from the TimeTree database. In these cases, we selected a node near the root of the phylogeny spanning two species present in TT5, then generated a divergence time estimated range from TT5 and treated this as a uniform time calibration for the study phylogeny. For large trees (10+ species), we added a second or third time-calibration near the present. All divergence time estimates were generated using RelTime in MEGA 11 (Tamura et al., 2012). We then added missing species to the backbone using the same approach as noted above.

We inferred molecular phylogenies for species that had never been included in molecular phylogeny, for which only genetic sequence data exist for a few genes. In these cases, we accessed the sequence data for each species, which was typically restricted to a single common gene (Supplementary Material for accession numbers). Then we performed an NCBI BLAST search to identify similar sequences. We selected up to three sequences from the congeneric species with the most similar BLAST E-values to the target sequence which were present in our backbone tree. We then selected an outgroup taxon, which we defined as a member of the Catarrhini for missing species that represented the Platyrrhini, and *vice versa*. The chosen set of sequences was aligned using MUSCLE in MEGA and a maximum likelihood phylogeny was constructed with 100 bootstrap replicates in sites with less than 50% data coverage across species were eliminated. These phylogenies were then time-calibrated using RelTime as described above, and the divergence times for each missing species were incorporated into our backbone tree as above. In three cases (*Cebus versicolor*, *Mico intermedius*, and *Presbytis senex*), taxonomic uncertainty precluded the designation of a consensus sister species, so we placed the new species at the base of the genus. Future work may seek to use morphological data to resolve such taxonomic uncertainty, but such analyses are outside the scope of the present study.

### 4.4 Macroevolutionary analyses

We calculated evolutionary isolation (EI) metrics using *picante* (Kembel et al., 2010) in R (Core Development Team, 2020) for our expanded timetree (eTT) and several others. Because the crown age of the eTT (42.2 myr) and that of the VertLife phylogeny (32.7 myr) differ substantially, we divided all observed EI values by the crown age before directly comparing them. This discordance in crown times is likely due to the effect of differing calibration schemes (Hedges et al., 2018). Because our eTT is calibrated based on literature consensus times, it may represent a better representation of the field as a whole than any given calibration scheme used in an individual publication.

We calculated the clade-wide speciation rate of apes and monkeys in TESS (Höhna et al., 2016). For this analysis, we specified 306 of an assumed 308 total species and allowed the model to infer hyperparameters of speciation (0.29) and extinction (0.08) as priors. We then ran the model for a total of 200,000 iterations setting aside 10,000 as burn-in. In this case, we did not choose to parameterize any historical mass extinction events, accepting the default parameters. Critical BayesFactors for the inference of rate shifts were set at 2, 6, and 10, but with the exception of the terminal rate shift, which may be ascribed to known systematic biases arising from phylogenetic incompleteness (Craig et al., 2022), no shifts were recovered.

## Data Availability

The original contributions presented in the study are included in the article/Supplementary Material, further inquiries can be directed to the corresponding author.
